# Advances in Drug Delivery via Biodegradable Ureteral Stent for the Treatment of Upper Tract Urothelial Carcinoma

**DOI:** 10.3389/fphar.2020.00224

**Published:** 2020-03-17

**Authors:** Hongli Shan, Zhongshuai Cao, Changliang Chi, Jixue Wang, Xiaoqing Wang, Jingyan Tian, Bing Yu

**Affiliations:** ^1^Department of Clinical Laboratory, The First Hospital of Jilin University, Changchun, China; ^2^Department of Urology, The First Hospital of Jilin University, Changchun, China

**Keywords:** local drug delivery, ureteral stent, chemotherapy, biodegradable, upper tract urothelial carcinoma

## Abstract

Drug eluting ureteral stent is an effective means for local drug delivery to the urinary tract. It can potentially solve a variety of upper urinary tract problems, such as stent-related urinary tract infections and discomfort, ureteral stricture, and neoplastic diseases. However, the release of drug elutes on the surface of biostable stents is unsustainable and uncontrollable. With the development of biomaterial science, the emergence of biodegradable ureteral stents (BUSs) provides a new approach for local drug delivery in the urinary tract. The drugs can be continuously released in a controlled manner from a drug-eluting BUS, when the stent degrades. Especially for the delivery of anti-tumor drugs, the stents can obviously improve the therapeutic effectiveness of the drugs by prolonging the contact duration of the drug and tumor cells. In addition, a secondary stent removal procedure can be avoided. The purpose of this review article is to provide an overview of anti-tumor drug-eluting BUSs and discuss the biomaterials and drug delivery systems of BUS that are currently being developed to deliver anti-tumor drugs for upper tract urothelial carcinoma.

## Introduction

For patients with low-risk upper tract urothelial carcinoma (UTUC), kidney-sparing surgery is recommended as the preferred treatment by the European Association of Urology (EAU) guidelines (Roupret et al., 2018). After kidney-sparing surgery, the recurrence of carcinoma and the side effects caused by indwelling ureteral stent were the main urgent problems to be solved (Zigeuner and Pummer, 2008). Local chemotherapy can obviously avoid the recurrence of carcinoma, which has been used as a routine adjuvant treatment after UTUC (Babjuk et al., 2017). However, due to the characteristics of impermeable urothelium and the continuous washing of urine, the effectiveness of local chemotherapy was limited. Therefore, it is needed to realize the controlled and sustained drug release in the upper tract, which can overcome the above problems (Mosayyebi et al., 2018).

With the rapid development of nanodrug loading technology, many localized cancers can be treated by direct deposition of nanoparticle-based drugs in the target area (Ji and Kohane, 2019). Some local nanodrug loading systems have been developed and become powerful tools to reduce recurrence and progression of non-muscle invasive bladder cancer in the past decades (Guo et al., 2019). For UTUC, the anti-tumor drug-loaded biodegradable ureteral stent might be an optional and effective method to solve the clinical problems. Incorporating coating and eluting as drug loading technologies with multiple advantages in the design of drug-loaded stents is not fully studied. In the field of cardiovascular disease, the drug-eluting degradable stents have been widely designed and studied (Waksman and Pakala, 2010; Wang et al., 2014). In addition, engineered nanomedicines have been reported to enhance the tumor penetration in local chemotherapy (Ding et al., 2019a). However, the researches of drug-eluting biodegradable stents in the urinary system are still scarce. In our review, we summarize the studies about drug-eluting biodegradable ureteral stents (BUSs) and discuss biomaterials and drug delivery systems of BUS that are currently being developed in delivering anti-tumor drugs.

## Biomaterials for BUS

The degradable drug-loaded ureteral stents have been getting more and more attention because of their advantages of no need for cystoscopy, no “forgotten stent” problem, and low incidence of infection and hematuria (Beysens and Tailly, 2018). The biomaterials used in BUS should be biocompatible, and have moderate mechanical strength to maintain intact tubular structure and well-controlled degradation rate, such as n polymers in nature, synthetic polymers, and some metals.

### Natural Origin Polymers

There are various polymeric biodegradable materials in nature with great potential to be selected as a material of BUS, such as collagen, gelatin, alginate, fibrin, silk, and so on. Their own characteristics, such as biocompatibility, microstructure, mechanical strength, degradation rate, and the effect of anti-infection should be carefully considered in the design of BUS. It is fortunate that many natural origin polymers can meet these conditions and have been widely used in design of BUS (Auge et al., 2002; Nady and Kandil, 2018). Lingeman et al. (2003) designed proprietary alginate polymer-based BUS and applied in 88 patients. This stent could maintain intact within 48 h and begin to degrade after that. The *in vivo* results indicated that the alginate-based stent could effectively facilitate the urinary drainage and exhibited excellent biocompatibility. It is noted that there was a 3.4% insufficient degradation of the stent, which needed a second intervention to remove the fragments. Barros et al. (2015b) fabricated a hollow ureteral stent, consisting of alginate, gellan gum, and templated gelation. Moreover, critical point carbon dioxide drying was used in the process of building. The *in vivo* degraded experiment indicated that this stent exhibited the fastest biodegradation rate than the control group. Moreover, the stent could effectively reduce the bacterial adhesion and showed excellent biocompatibility properties (Barros et al., 2018).

### Synthetic Polymers

Over the last decades, synthetic polymers have been designed to fabricate biodegradable drug carriers and are widely applied in tissue engineering, nanodrug delivery systems, and diagnosis of disease. Various kinds of synthetic polymers have been used to prepare matrix and implant forms because of their distinctive advantages, such as excellent biocompatibility, controlled biodegradation rate, ideal mechanical strength, and hydrophobicity/hydrophilicity (Kim et al., 2014; Kapoor et al., 2015). Alpha hydroxy acids, such as polylactic acid (PLA), polyglycolic acid, poly (lactide-co-glycolide) (PLGA), polycaprolactone (PCL), polyethylene glycol, poly (lactide-co-caprolactone), polydioxanone, etc., were the most commonly used synthetic polymers (Wang L. et al., 2018). Moreover, it is noted that all these polymers were approved by the U.S. Food and Drug Administration (FDA) as biomaterials (Wang et al., 2015b) and Lumiaho et al. (2007) fabricated a biodegradable ureteral stent by using PLA as the basic material. After implanting in porcine model, the results indicated that the ureteral stent exhibited satisfactory drainage characteristics and excellent antireflux effect. However, the stent degraded in blocks and caused poor drainage of ureter. Uriprene, a novel biodegradable stent constructed by PLGA, was designed to gradually degrade from distal to proximal end to avoid ureteral obstruction by fragments. Moreover, a third-generation stent that can degrade in a short time has also been designed. The *in vivo* results indicated that this stent could degrade by 90% within 28 days and played excellent drainage effect (Chew et al., 2010, 2013). The authors of this review fabricated a nanostructured PLGA ureteral stent by double-needle electrospinning. The *in vivo* animal experiment exhibited that the stent could completely degrade within 10 weeks post-insertion, and degrade from distal to proximal. Moreover, compared to a commercial stent, this stent showed less impact on epithelial cells (Wang et al., 2015a).

### Metals

In terms of mechanical strength, metallic stents have obvious advantages over polymer counterparts. Some metals are suitable for fabricating biodegradable stents since they are what the body contains itself (Chew et al., 2010, 2013). For example, magnesium (Mg) is suitable for constructing medical devices due to its lightweight and biodegradable properties, especially in the cardiovascular field. In the urology field, Mg-based alloy exhibited excellent biodegradability and could effectively inhibit the growth of bacteria.

Lock et al. (2014) evaluated the antibacterial and biodegradable properties of magnesium and its alloys for potential biodegradable ureteral stent applications. The results showed that magnesium alloys decreased *E. coli* viability and reduced the colony formation units in an artificial urine solution when compared with commercial polyurethane stent.

Tian et al. (2019) investigated the cytocompatibility and degradation behaviors of four promising Mg-based alloys *in vitro*. The results indicated that the degradation rates of these Mg-based alloys should be further reduced for the sake of reducing the side effects of the soluble or insoluble degradation substances.

## Anti-Tumor Drug-Eluting BUS

With the rapid development of nanotechnology, nanodrug delivery systems have been widely researched to delivery therapeutic agents, which could efficiently improve the delivery efficacy (Chen et al., 2017; Wang J. et al., 2018). For non-muscle invasive bladder cancer, one effective way to prevent cancer recurrence is intravesical instillation chemotherapeutic drugs after transurethral resection of the bladder cancer (Babjuk et al., 2017). Because of the same biological characteristics as bladder cancer, UTUC could also be treated by local chemotherapy. However, the particularity of the upper urinary tract anatomy limited the application of local chemotherapy and there are currently no effective local chemotherapy methods.

The biodegradable anti-tumor drug-loaded ureteral stents had been widely researched and had been demonstrated that they could improve the situation of chemotherapy of UTUC. It can last for a long-term effective loaded anti-tumor agent release in the ureter when the stent degrades. The drugs could permeate through the ureter, effectively affecting the cancer cells. It may also solve the problems of secondary removal simultaneously. However, reports on the application of drug-eluting techniques for biodegradable stents in urinary system are limited.

Barros et al. (2016) fabricated a biodegradable ureteral stent, which was impregnated by supercritical fluid CO_2_. Furthermore, four different kinds of anti-tumor agents, including paclitaxel, doxorubicin, epirubicin (EPI), and gemcitabine, were loaded into the stent. The *in vitro* anti-tumor experiment indicated that these drug-eluted stents could efficiently suppress the growth of urothelial cancer cell (T24 cells). In addition, all these drug-eluted stents showed minimal toxicity toward the non-cancer cells (HUVEC cells). These results indicated that the impregnated BUSs could be used as proper anti-tumor drug carriers and potentially be an effective intravesical drug delivery system for UTUC therapy. In their next study, the researchers used different membranes to research the permeability of the loaded anti-tumor agents, including single paclitaxel and doxorubicin, and their release from the prepared BUS. The results demonstrated that the release performances of paclitaxel and doxorubicin from the BUS could be kept for a long time in the *ex vivo* ureter and only a small amount of the drugs can across the different permeable membranes with a permeability of 3% for paclitaxel and 11% for doxorubicin. All these results demonstrated that most drugs in these BUS could remain in the *ex vivo* ureter tissue, which were effective to suppress the growth of tumor cells and not affect the non-tumor cells (Barros et al., 2017). We also fabricated an anti-tumor drug-loaded biodegradable ureteral stent to suppress the recurrence of UTUC after kidney-sparing surgery (Wang et al., 2019). In our research, different kinds of degradable PCL/PLGA scaffolds consisting of different proportions of PCL and loading EPI were prepared by electrospinning. The results indicated that the PCL/PLGA scaffolds could sustain the release of loaded drugs and exhibited controlled degradation. Moreover, the drug release and degradation rates of scaffolds were slowed down with the increase of PCL. The anti-tumor activity demonstrated that the scaffolds could efficiently suppress the growth of bladder tumor cells both *in vitro* and *in vivo*. Moreover, their *in vivo* application showed no apparent systemic toxicity. Our results demonstrated that these electrospun polyester scaffolds could be effectively used for local inhibition of recurrence of UTUC after surgery.

## Design of Drug Delivery System for BUS

Compared with systematic administration, *in situ* administration can effectively improve the drug concentration at the targeted sites. In local drug delivery of upper urinary tract, the key goal is to keep an effective drug treatment concentration and long-term maintenance at upper urinary tract. The second goal is to reduce the effect of drugs on non-tumor tissues, so as to reduce the toxicity of drugs to the body. The particular local anatomy and microenvironment of the upper urinary tract are potential barriers to local delivery (Weiser and Saltzman, 2014; Mittal et al., 2018). Some previous researches had coated drugs to the surface of biostable stents, and the results were not satisfactory due to the uncontrolled drug release. However, drug-eluting BUS could sustain a long-term effective drug release during the process of stent degradation, and at the same time avoid a secondary removal procedure. There are many drug delivery systems for loading drugs, including hot melt extrusion, soaking the polymers into drug solution, CO_2_ impregnation, nanofibers, and nanoparticles.

### Traditional Drug Delivery Systems

Drug delivery systems, such as hot melt extrusion, solvent-casting, and soaking the polymers into a drug solution, are traditional drug delivery systems. It contains numerous limitations, such as bad drug control release, lack of targeting, poor water solubility, and susceptibility to drug resistance (Tiwari et al., 2012; Edgar and Wang, 2017; Pandit et al., 2017). Lim et al. (2018) designed a bilayer swellable drug-eluting ureteral stent used for treating urothelial diseases. The drug mitomycin C was directly mixed with lactide-co-caprolactone pellets and dissolved in dichloromethane. After that, the mixed solution was coated onto the surface of the ureteral stent. To achieve sustainable drug release at the ureteric diseased site, the researchers further coated a layer of hydrogel on the surface of the stent. The results indicated that the drug could be continuously delivered over 1 month. The *in vivo* results also demonstrated the improved drug release performance and the advantage in transporting into explanted porcine ureteric tissues under a simulated dynamic fluid flow.

### CO_2_ Impregnation

**FIGURE 1:**
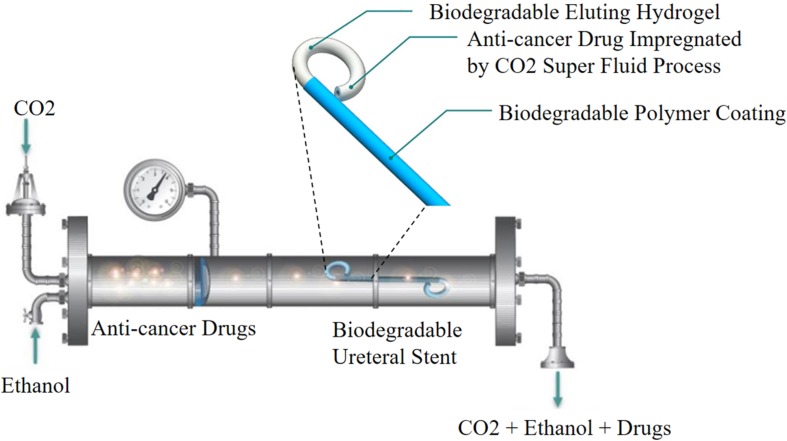
Schematic representation of CO_2_ impregnation drug-eluting BUS. Reprinted from Barros et al. (2016) under open access license.

The CO_2_ impregnation has attracted growing interest and applied in various fields, which allows loading a drug into an already manufactured implant. It is noted that CO_2_ in its supercritical state can dissolve well into various polymer matrices. In addition, it has low critical coordinates (Champeau et al., 2015). Barros et al. (2015a) first used this technique in preparing a ketoprofen-eluting BUS. These BUSs were fabricated by alginate or gellan gum–based polymers, and the stent was impregnated with ketoprofen. The results showed the release of ketoprofen in the first 72 h was very promising, which was in accordance with the time needed for anti-inflammatory treatment after operation. The *in vitro* release results indicated that the temperature had an obvious effect on the impregnation yield. The same research group also used the same methods to load paclitaxel and doxorubicin into a natural origin polymer-based BUS for the treatment of UTUC. The results indicated that the release of paclitaxel and doxorubicin from the BUS could affect the tumor cells and not affect the non-tumor cells (Barros et al., 2016). However, the impregnation process also had some drawbacks, including the drug must have sufficient solubility in CO_2_, low drug loading rate and drug loading efficiency, and the stent shape changes after drug loading (Figure 1; Ugaonkar et al., 2011; Champeau et al., 2015).

### Electrospun Nanofibers

Electrospinning can converse polymeric solution/melt into solid nanofibers through the application of electrical force. The drug-loaded electrospun nanofibers have been getting more and more attention because of their distinctive properties, including high encapsulation efficiency for drug loading, controlled residence time, desirable delivery of encapsulated drug at a predictable rate, better stability, high surface contact area, degradability, and satisfactory softness and flexibility (Zamani et al., 2013; Ding et al., 2019c). Moreover, electrospun nanofibers have a nanosized diameter and a structure mimicking that of extracellular matrix proteins. Interestingly, the occurrence of degradation of extracellular matrix proteins also plays an important role in tumor metastasis (Alizadeh et al., 2018; Feng et al., 2019a). Therefore, the local and continuous delivery of chemotherapy agents can improve the concentration of agents in tumor tissue, delay and control the drug release at cytotoxic concentrations, thus significantly reducing the toxicity to normal tissues of the body, reducing the frequency of drug use, and avoiding the damage to normal vascular endothelium. Li et al. (2018) developed a local drug delivery system consisting of an emulsion-electrospun polymer patch for the treatment of primary and advanced orthotopic hepatomas. The anti-tumor drugs could realize sustained release, and could efficiently suppress the tumor growth and invasiveness in animal models.

The authors of this review first used two types of electrospinning technique to develop a series of epirubicin (EPI)-loaded PCL/PLGA nanofiber ureteral stent with adjustable rates of drug release and degradation for local chemotherapy of UTUC. First, the EPI-loaded PCL/PLGA fibers were developed by general electrospinning technique, and the drug was dispersed in the fibers, both the inside and the surface. The nanofibers showed sustained drug release and controlled degradation, and satisfactory anti-tumor effects both *in vitro* and *in vivo*. Moreover, all of them exhibited no apparent systemic toxicity (Wang et al., 2019).

**FIGURE 2:**
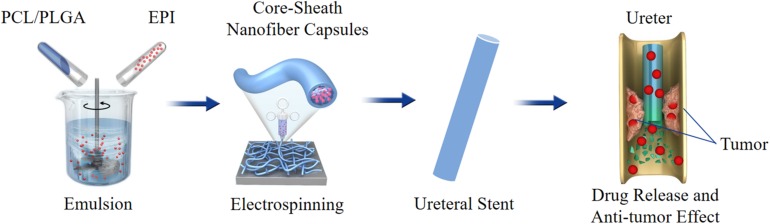
Schematic illustration of preparation and antitumor effect of EPI-loaded BUS.

However, early drug release of the fibers was relatively fast, which may affect anti-tumor effects. Emulsion-electrospinning technology was used to generated a core–sheath structured EPI-loaded PCL/PLGA nanofiber capsules. The core–sheath structures of these fibers were confirmed by a confocal laser scanning microscope, and the EPI was loaded in the inner layer of the fiber. The EPI release was more sustained than that of fibers generated by general electrospinning. The nanofiber capsules effectively inhibited the growth of tumor cells both *in vitro* and *in vivo*, with no apparent systemic toxicity (Figure 2; Sun et al., 2019). Although nanofibers have exhibited great potential in drug delivering, before using this technology as a mainstream drug delivery method, they still face some challenges, including low drug loading efficiency, instability of active ingredients, initial sudden release of drugs, number of residual solvents, and industrialization (Luo et al., 2012; Thakkar and Misra, 2017).

### Nanoparticle-Based Drug Delivery Systems

Nanoparticle-based drug delivery systems have been widely used as an effective means for local drug-controlled release systems, and have been widely used in various fields of biomedicine (Feng et al., 2019b; He et al., 2019). Various nanoparticle-based drug delivery systems, such as liposomes, nanogels, self-assembling peptides, micelles, water-soluble polymers, and carbon nanotubes have been successfully applied to targeted treatment of cancer. Nanoparticle-based drug carriers can improve the therapeutic effects of the loaded drugs by using active targeting for the site-specific delivery, and protecting normal cells from damage (Park, 2014; Ashfaq et al., 2017).

Because of the particularity of urinary tract diseases, there are many challenges to realize *in situ* delivery of drugs. First, the dilution and washing of urine reduce the bioavailability of drugs in the urinary tract. In addition, the limited drug exposure time will influence the treatment effect. Furthermore, when drugs enter tumor cells, they will be blocked by high resistance and tight connection of drugs into superficial cells, and by high resistance and tight connection of superficial cells and semirigid and asymmetric urothelial membrane (Tyagi et al., 2016; Mittal et al., 2018). In order to overcome these problems, higher doses of drugs are usually needed to achieve effective therapeutic concentration in the target tissue. Nanoparticle-based drug delivery systems with cytotoxic agents of favorable size and capable of adhering to urothelium can potentially prolong the duration of action and decrease toxicity (Tyagi et al., 2016; Ding et al., 2019b).

**FIGURE 3:**
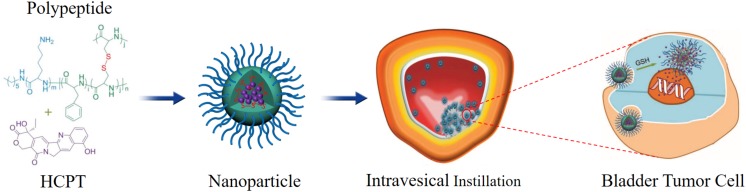
Schematic illustration of chemical structure of polypeptide/HCPT nanoparticles and its metabolic process. Reproduced from Guo et al. (2018) under open access license.

Erdogar et al. (2014) designed a chitosan- and PCL-based core–shell nanoparticles to deliver mitomycin C, and evaluated the anti-tumor efficacy of the delivery system in bladder tumor model. The results indicated that the drug-loaded nanoparticles treatment group showed the longest survival rate compared to other groups. Histopathological results indicated that the cationic nanoparticles were mainly localized and accumulated in the bladder tissue, and no mitomycin C was quantified in blood. In another study, the delivery of docetaxel to bladder tumor was enhanced by mucoadhesive nanoparticles formed by hyperbranched polyglycerols. Nanoparticles improved the permeability of docetaxel across urothelium and enhanced the uptake into the animal tumor cells (Mugabe et al., 2011). Guo et al. (2018) designed a novel disulfide-cross linked polypeptide nanoparticles of poly (l-lysine)-poly (l-phenylalanine-co-l-cystine) to efficiently deliver HCPT to treat orthotopic bladder tumor. The results indicated that these positively charged HCPT-loaded nanoparticles could efficiently adhere to the negatively charged tumor cell membrane and internalized into the cytoplasm through the electrostatic interaction. After that, the high concentration of glutathione in bladder tumor cells could efficiently trigger the cleavage of the disulfide bond, which caused the fast release of loaded HCPT. The results showed that this nanosystem could efficiently improve the retention time and enhance the tumor tissue permeability of HCPT (Figure 3).

**FIGURE 4:**
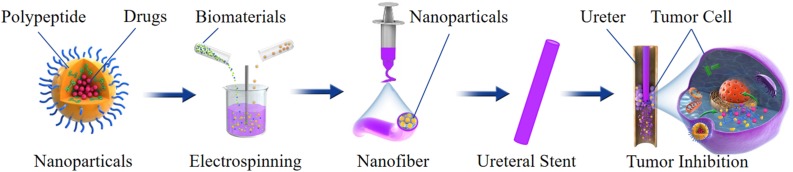
Schematic illustration of preparation, structure and antitumor effect of a nanoparticles-based drug-eluting BUS.

Based on the above technology, the nanoparticles can also be loaded on the BUS to deliver anti-tumor drugs. For example, an anti-tumor drug can be loaded into polypeptide nanoparticles; then, the drug-loaded nanoparticles are mixed with the biomaterials to fabricate ureteral stents by electrospinning. With the degradation of the ureteral stent, the nanoparticles will continuously release drugs; it can extend the exposure time and enhance the penetration of the nanoparticles into the urothelium. When the nanoparticles are phagocytosed by cells, endogenous stimuli cause the structural changes of the nanoparticles, and the drug will be rapidly released in the cells, which could improve the drug delivery efficiency and reduce toxicity (Figure 4).

## Conclusion

The use of drug-eluting BUS in urology clinical practice not only provides local chemotherapy but also opens new treatment options. Various advancements in biomaterials, design, and drug delivery systems of BUS in this paper are reviewed. With the most appropriate biomaterial, design, and drug delivery technique, an optimal anti-tumor drug delivery stent could be developed in the future. However, there is no ideal stent, and we wish that our review can provide a summary of the technological challenges that need to be overcome in the development of constructing drug-eluted BUS. Future research should focus on how to use nanoparticle-based BUS to deliver local treatment drugs effectively.

## Author Contributions

XW, JT, and BY: conceptualization. HS, ZC, CC, and BY: literature and data review. HS and JW: writing – original draft. XW and JT: writing – review and editing.

## Conflict of Interest

The authors declare that the research was conducted in the absence of any commercial or financial relationships that could be construed as a potential conflict of interest.
